# The Salmon Louse *Lepeophtheirus* *salmonis* (Copepoda: Caligidae) Life Cycle Has Only Two Chalimus Stages

**DOI:** 10.1371/journal.pone.0073539

**Published:** 2013-09-12

**Authors:** Lars A. Hamre, Christiane Eichner, Christopher Marlowe A. Caipang, Sussie T. Dalvin, James E. Bron, Frank Nilsen, Geoff Boxshall, Rasmus Skern-Mauritzen

**Affiliations:** 1 Sea Lice Research Centre, University of Bergen, Bergen, Norway; 2 Aquatic pathogens and diseases, Institute of Marine Research, Bergen, Norway; 3 Institute of Aquaculture, University of Stirling, Stirling, Scotland, United Kingdom; 4 Natural History Museum, London, United Kingdom; University of Toronto, Canada

## Abstract

Each year the salmon louse ( *Lepeophtheirus* *salmonis* Krøyer, 1838) causes multi-million dollar commercial losses to the salmon farming industry world-wide, and strict lice control regimes have been put in place to reduce the release of salmon louse larvae from aquaculture facilities into the environment. For half a century, the *Lepeophtheirus* life cycle has been regarded as the only copepod life cycle including 8 post-nauplius instars as confirmed in four different species, including *L* *. salmonis* . Here we prove that the accepted life cycle of the salmon louse is wrong. By observations of chalimus larvae molting in incubators and by morphometric cluster analysis, we show that there are only two chalimus instars: chalimus 1 (comprising the former chalimus I and II stages which are not separated by a molt) and chalimus 2 (the former chalimus III and IV stages which are not separated by a molt). Consequently the salmon louse life cycle has only six post-nauplius instars, as in other genera of caligid sea lice and copepods in general. These findings are of fundamental importance in experimental studies as well as for interpretation of salmon louse biology and for control and management of this economically important parasite.

## Introduction

Sealice (members of the copepod family Caligidae) are a major health hazard for farmed finfish and the salmon louse, *Lepeophtheirus* *salmonis* (Krøyer, 1838) alone is responsible for commercial losses in excess of 180 million € in salmonid aquaculture in the Northern Hemisphere [1].

The complete life cycle is now known for 17 species of Caligidae representing just three genera, Caligus Müller 1785 (12 species), Lepeophtheirus von Nordmann 1832 (four species) and *Pseudocaligus* A. Scott 1901 (one species) [2,3]. The number of stages described for the free-living phase is common to all *Caligus*, *Pseudocaligus* and *Lepeophtheirus*: two nauplii and the infective copepodid stage, but the number of stages between the infective copepodid and the adult appears to vary [4]. Following attachment to the host the copepodid molts into the first of a number of chalimus stages, which are characterized by possession of a frontal filament that firmly secures their attachment to the host. In *Caligus* and *Pseudocaligus* species four chalimus stages are found, the last of which molts into the definitive adult (e.g. [2,5,6]). In contrast, the life cycle of *Lepeophtheirus* species has been reported to comprise four chalimus plus two preadult stages [7,8,9,10], with preadults being distinguished from chalimi by their ability to detach from a temporary frontal filament shortly after molting [11] and move over the surface of the host.

There have been occasional reports of more than four chalimus stages in *Caligus*: for example, a total of six chalimus stages was reported in *C* *. epidemicus* Hewitt, 1971 [12], but subsequent reinterpretation revealed only four molt stages (see 2). Reports of pre-adult stages in life cycles of *Caligus* species such as *C* *. spinosus* Yamaguti, 1939 [13] and *C* *. clemensi* Parker & Margolis, 1964 [14], similarly failed to stand up to detailed scrutiny. Ho and Lin [4] concluded that so-called preadults described in *Caligus* species are freshly-molted, young adults. Development in *Pseudocaligusfugu* Yamaguti, 1936 follows the same pattern as in *Caligus*, with a total of eight stages: two nauplius, one copepodid and four chalimus stages preceding the adult, without any preadult stage [2].

All free-living copepods for which the life cycles are known have six post nauplius stages collectively referred to as copepodid stages among copepodologists. *Caligus* and the vast majority of parasitic copepods also have six copepodid stages in total. In fact, *Lepeophtheirus* is the only genus in the entire Copepoda for which the number of copepodid stages is reported to exceed six. Species of this genus are reported to have a life cycle comprising ten stages in total: two nauplius plus eight copepodid stages; the infective copepodid, four chalimus stages, two pre-adults and the adult [7,8,9]. It seemed anomalous that this one genus should have two additional stages in the copepodid phase of its life cycle, so Ohtsuka et al. [2] reconsidered the life cycle of *Lepeophtheirus*, asking whether all the identified stages in the life cycle were separated by molts. Antennulary setation strongly indicates stage-specific developmental patterns in copepods [15] and using the segmental setation pattern of the caligid antennules as an indicator of stage identity, Ohtsuka et al. [2] concluded that “in *L* *. salmonis* and *L* *. pectoralis* (O.F. Müller, 1776) there are two chalimus stages which require confirmation as true instars: chalimus II only differs from chalimus I in size and in the degree of development of certain limbs, and chalimus IV only differs from chalimus III in the same way, not in setal numbers [8,9]. It is conceivable that these paired stages (chalimus I-II and chalimus III-IV) represent only intramolt growth stages and, given the commercial importance of sea lice, this should be tested”. The hypothesis that chalimus I-II and chalimus III-IV represent intramolt variation of only two chalimus stages has been further strengthened in a recent paper on *Lepeophtheirus* *elegans* [16].

While molting in the free living stages can be observed directly and molts between preadult 2 and adult lice can be directly observed on the fish host ( [11], unpublished observations), molts between the different chalimus stages have not been observed previously. We have observed that *L* *. salmonis* copepodids, chalimi and preadults are able to molt off the host when maintained in incubators leaving observable exuviae (unpublished observations). In the present study we investigate the number of chalimus stages in the *L* *. salmonis* life cycle by observing molts in incubators and by identifying the number of distinct chalimus size groups.

## Materials and Methods

Two similar but independent experiments were conducted at the Sea Lice Research Centre (Experiment 1) and the Institute of Marine Research in Bergen (Experiment 2) to determine the number of chalimus stages in *L* *. salmonis* . Experiment 1 used observations of abandoned *L* *. salmonis* exuviae in incubators to identify molting events prior to the preadult 1 stage. In experiment 2 morphometrics were used to identify the number of distinct morphological groups of *L* *. salmonis* chalimus larvae and to assess molting in incubators.

In both experiments Atlantic salmon (*Salmo salar*) were stocked in 500L fish tanks (1x1x0.5 m) with flow-through full salinity seawater (34.5‰, 10±0.3°C). The fish were infected with *L* *. salmonis* copepodids as described by Hamre et al. [17] using 150-200 copepodids fish^-1^. The lice used belong to the laboratory strains LsGulen and LsOslofjord [17,18]. Both experiments were carried out in strict accordance with Norwegian legislation and the experiment was approved by the Norwegian Animal Research Authority (permits nr. 2010/245410 and 2009/186329)**.**

### Sampling of *L* *. salmonis*

**Table 1:** Number of *Lepeophtheirus* *salmonis* sampled.

	Experiment 1		Experiment 2		
DPI	ch I-II	ch III-IV	ch I-II	ch III-IV	preadult 1
5	ns	ns	48/13/0	0	0
6	30/30/0	0	ns	ns	ns
7	38/38/0	0	7/7/0	0	0
8	29/29/1	0	4/2/0	0	0
9	54/54/18	0	7/0/0	ns	ns
10	25/25/15	14/14/0	95/30/12	8/0/0	0
11	14/14/10	34/34/0	57/57/15	ns	ns
12	3/3/2	53/53/0	5/5/2	50/3/0	0
13	4/4/1	85/85/18	ns	ns	ns
14	1/1/1	81/81/28	2/2/0	108/58/12	1/0/0
15	0	46/46/21	ns	ns	ns
16	0	46/46/35	0	76/29/10	42/0/0
17	0	68/68/52	0	35/35/19	ns

Number of *L* *. salmonis* sampled, incubated and molted in experiment 1 and 2 respectively. The numbers are given as a/b/c where a= larvae sampled, b= larvae stocked in incubators and c= observed molts as defined in the text Ch I-II =larvae identified as either chalimus I or II, Ch III-IV=larvae identified as either chalimus III or IV (as defined by Johnson and Albright [8] and Schram [19]). DPI=days post infection at sampling, ns=not sampled.

Starting at 5 days post infection (DPI) fish were sacrificed regularly in order to collect *L* *. salmonis* larvae. The detailed sampling scheme is given in Table 1. The fish were killed by a blow to the head and any stages of *L* *. salmonis* present were gently removed and photographed upon removal for morphometric measurements. In both experiments and at all sample points, except for sampling at days 11 and 17 in experiment 2, great care was taken to remove all larvae to avoid biases in size and sex. Samplings at day 11 and 17 in experiment 2 were specifically intended to elucidate molting events and therefore only chalimus I/II (as defined by Johnson and Albright [8]) were sampled on day 11 and only chalimus III/IV (as defined by Johnson and Albright [8]) were sampled on day 17. The majority of the sampled chalimus larvae (details in Table 1) were stocked individually in small continuous flow incubators described in [17] or smaller 32mm versions of these (see www.SLRC.no). Incubators had flow-through full salinity seawater (34.5‰, 10±0.5°C). In experiment 1 a total of 625 chalimi were sampled and placed in incubators (Table 1). The chalimi were inspected daily for at least 4 days to check whether a molt had taken place as defined by presence of an exuvium in the incubator. About 1/3 of the incubators were observed daily for up to 6 days. Where a molt had occurred the larva was photographed again. In addition, some chalimi which did not molt were photographed and measured again.

In experiment 2 a total of 545 lice were sampled from the fish and a total of 241 chalimus larvae were individually placed in incubators (Table 1). The larvae were photographed before incubation and were kept in the hatching wells for one to four days. The larvae were inspected daily and the point of sampling was determined by comparing pre-incubation size (determined as described in section 2.2) to the size at observation using an ocular scale. Larvae that had increased in size upon inspection were sampled and photographed and all remaining larvae were sampled and photographed when terminating the incubation. Images were obtained using either a Nikon Digital Sight DS-5M camera mounted on an Olympus SZX9 dissecting microscope (experiment 1) or an Imaging MicroPublisher 5.0 camera mounted on an Olympus SZX10 dissecting microscope (experiment 2).

### Morphological measurements and stage determination

**Figure 1:**
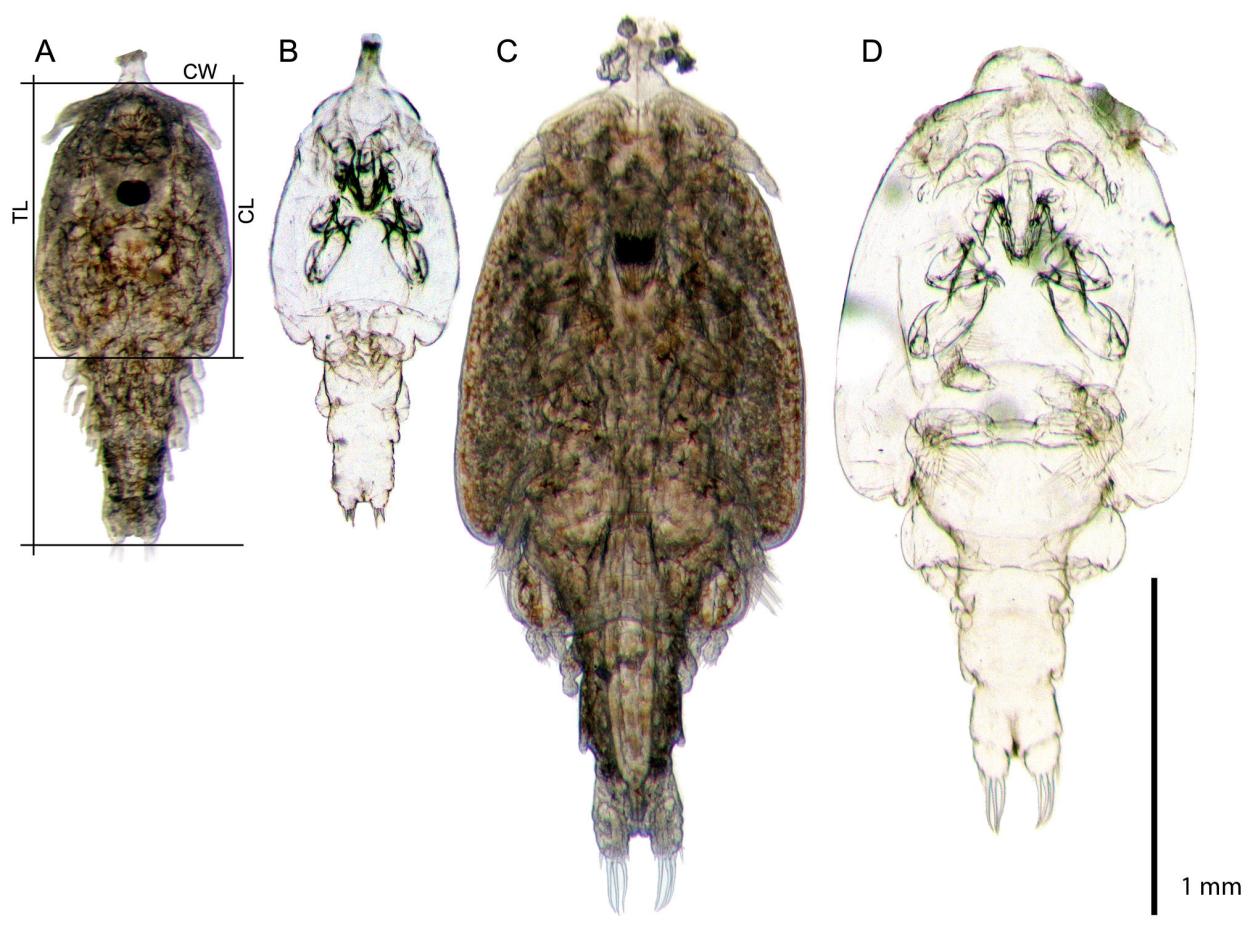
Chalimus larvae and shed exuviae. Chalimus larvae and shed exuviae belonging to the chalimus I/II category (A,B) and the chalimus III/IV category (C,D). The exuviae shown are the actual exuviae shed by the depicted larvae. The total length (TL), cephalothorax length (CL) and cephalothorax width (CW) measurements are shown in A.

Total length (TL) was measured for all samples. In addition, cephalothorax length (CL) and cephalothorax width (CW) were measured in experiment 2 (illustrated in Figure 1A). Measurements were made for all lice when they were removed from the fish (collectively referred to as pre-incubation measurements even if samples were not incubated in hatching wells). Post-incubation measurements were made for all incubated larvae in experiment 2 and only for those that molted in experiment 1 with some exceptions. All measurements were made using ImageJ v. 1.43 software. Due to image quality some lice were not measured.

Experience has shown that discrimination between chalimus I and II, and likewise between chalimus III and IV based on the morphological descriptions by Johnson and Albright [8] and Schram [19] is challenging. Therefore the samples were identified as preadults or assigned to a chalimus I/II or chalimus III/IV category as this level of determination could be achieved consistently. The number of distinct chalimus size groups was evaluated by means of cluster analysis (described below). The sex of chalimi that molted into preadults was determined based on preadult morphology [8,19].

### Molting identification

In experiment 1, molting was defined by the presence of a shed exuvium in the incubator. In experiment 2, molting was defined based on size increment between pre- and post incubation measurements. Data from experiment 1 showed that all confirmed molts were associated with TL increments >15% and this was used as a threshold to identify molts in experiment 2.

### Morphometric clustering

The chalimus samples from experiment 2 were clustered based on the morphometric data (TL, CL and CW) by K-mean clustering using the Hartigan and Wong algorithm and 10000 random starting seeds. A hierarchical clustering (based on Euclidian distance matrix and Ward’s minimum variance method) was also applied. However, as no hierarchical structure was expected in the data and the hierarchical clustering gave identical results, only the results of K-mean clustering are presented. Only pre-incubation measurements were used for clustering since growth may be reduced by the deprivation of food resulting from incubation in hatching wells as previously reported from similar experiments with *Panulirus* *cygnus* [20]. Since *L* *. salmonis* has been described to have four chalimus stages [8,19] the optimal number of clusters was selected based on the change in the proportion of unexplained variation (within groups sum of squares/total sum of squares) for one to four clusters. The separation of the selected number of clusters was tested by three separate ANOVA analyses using cluster number as predictor variable, TL, CL and CW respectively as response variables, and using a Bonferroni correction for the multiple tests to the significance values. Results were estimated as statistically significant when p ≤ 0.05/3, as 3 tests were conducted. All statistical and exploratory operations were performed in R v2.15.0.

## Results

**Figure 2:**
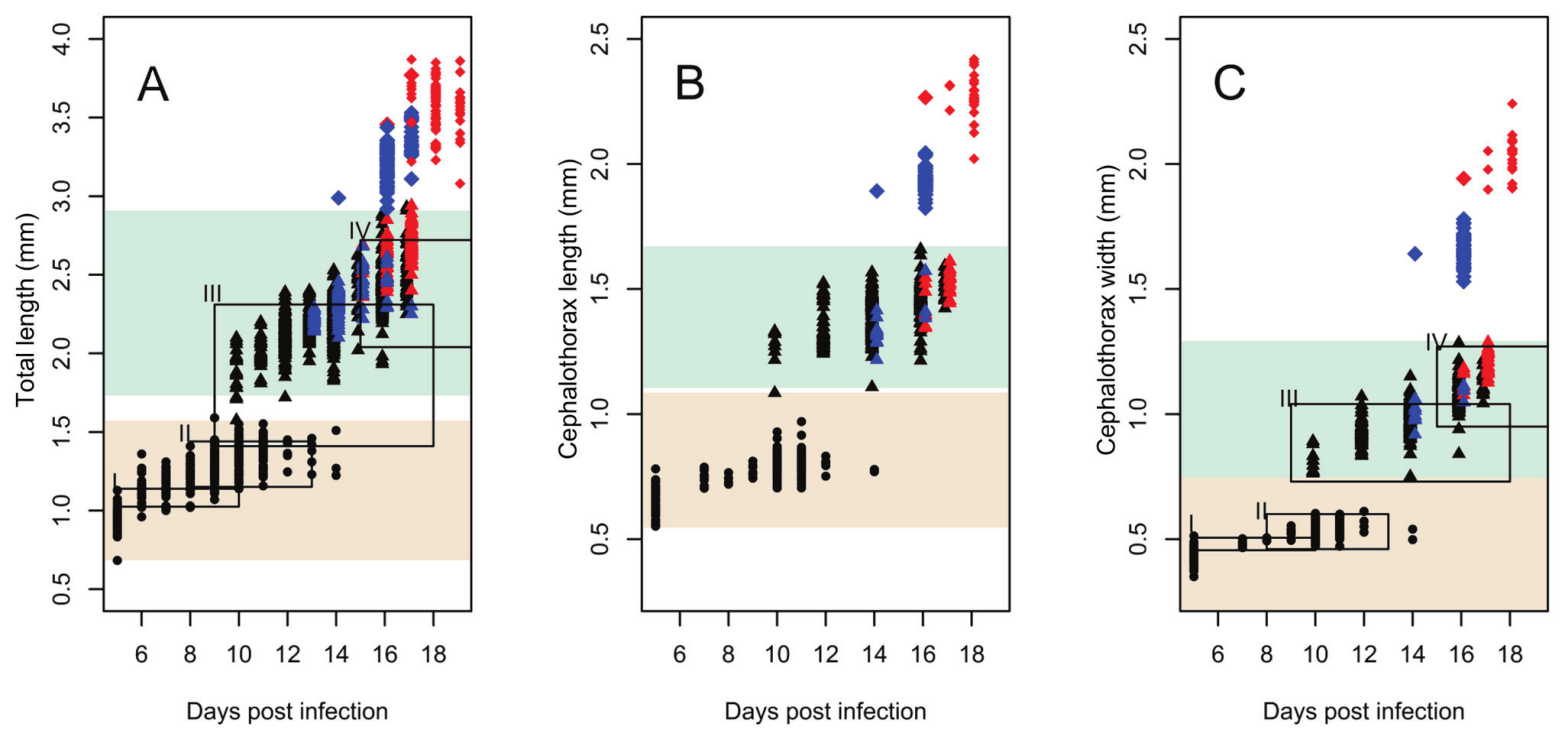
Increase in size of *L* *. salmonis* larvae post infection. Total length (TL) measurements from both experiment 1 and 2 are shown in A. Cephalothorax length (CL) and cephalothorax width (CW) measurements from experiment 2 are shown in B-C. Samples assigned to the chalimus I/II category are shown as circles. Chalimus larvae assigned to the chalimus III/IV are shown as triangles. Preadult 1 are shown as diamonds. Post-incubation measurements are not shown except for preadult I females shown as small diamonds. Color codes: black=sex not determined, blue=male, red=female. Red shaded area= size range of cluster 1, blue shaded area=size range of cluster 2. Frames I-IV represent the sizes for the chalimus I-IV stages as reported by Schram [19] and durations as reported by Johnson and Albright [28] (chalimus IV duration extends to 21 DPI). Size and duration boxes are not shown for CL as no data were reported.

**Figure 3:**
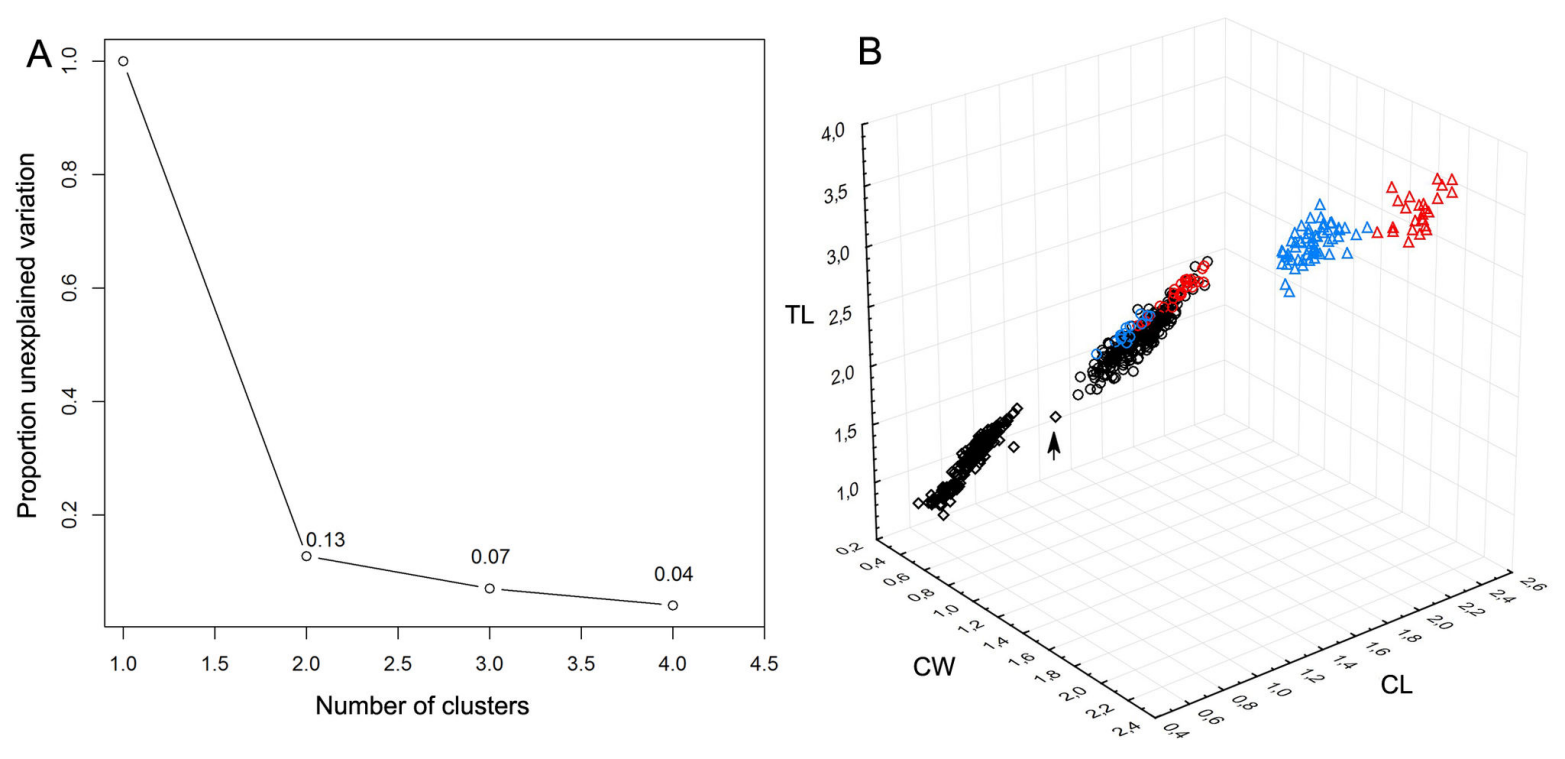
Cluster analysis and cluster number selection for chalimi. The proportion of unexplained variability in the dataset as a function of the number of chalimus clusters is shown in A. Total length (TL), cephalothorax length (CL) and cephalothorax width (CW) for experiment 2 (mm) are shown in B. Chalimus larvae assigned to chalimus cluster 1 are shown as diamonds. Chalimus larvae assigned to chalimus cluster 2 are shown as circles. Preadult 1samples are shown as triangles. Color codes: black=sex not determined, blue=male, red=female. Post-incubation measurements are not shown except for preadult I females (red triangles). The assignments to cluster (chalimus cluster 1 and 2) and morphological group (chalimus I/II, chalimus III/IV or preadult category) were congruent for all samples except the individual marked with an arrow.

Total length (TL), cephalothorax length (CL) and cephalothorax width (CW) of the sampled chalimi displayed discrete increments around 11 and 16 DPI (Figure 2 A-C). CL and CW appeared to be relatively stable between these increments, whereas a more continuous increase with threshold jumps at 11 and 16 DPI was evident in TL. The proportional reduction of unexplained variation with increasing numbers of clusters identified by K-mean clustering showed that attributing the chalimi to 2 clusters accounted for >87% of the variation in the morphometric dataset, whereas adding clusters beyond this number gave low proportional decrease in unexplained variation (Figure 3A). The two chalimus clusters (cluster 1 and 2) were significantly different in all morphometric dimensions (TL, CL and CW, ANOVA p-values < 0.0001) as illustrated in Figure 3B. Chalimus cluster 1 contained the 226 smallest individuals (Figures 2 and 3B) of which 225 were assigned to the chalimus I/II. The last chalimus in chalimus cluster 1was the smallest chalimus III/IV individual in terms of TL and CL and the second smallest in terms of CW (Figure 3B, marked with arrow). Chalimus cluster 2 comprised the 276 largest chalimus larvae that were all identified as chalimus III/IV (Figures 2 and 3B). The size ranges of the clusters showed a slight overlap of CW between cluster 1 and 2 (caused by a single individual shown in Figure 3B) whereas the morphologically determined chalimus I/II and III/IV categories did not overlap in chalimus size ranges (Table 2).

**Table 2:** Size range (mm) of the sampled chalimus categories.

Category	TL range	Sample size (n) TL	CL range	CW range	Sample size (n) CL and CW
Chalimus I/II	0.88-1.50	429	0.62-0.87	0.37-0.60	225
Chalimus III/IV	1.58-2.94	676	1.09-1.70	0.76-1.22	277

**Figure 4:**
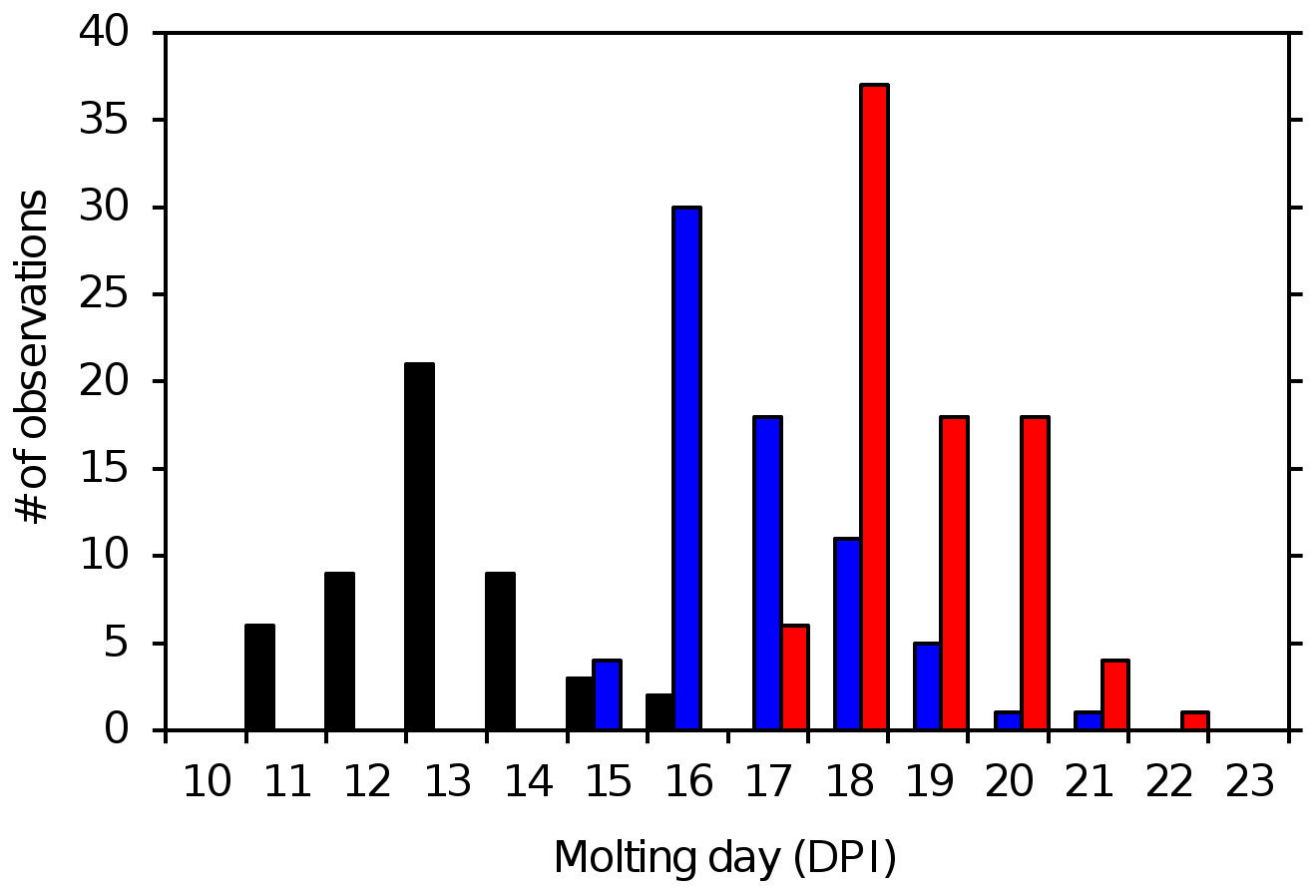
Time of molting. Time of molting in incubators for chalimus I/II and chalimus III/IV larvae. The presence of a shed exuvium was used as evidence that a molt had taken place within the past 24 h. Chalimus I/II=black, chalimus III/IV males=blue, chalimus III/IV females=red. DPI= days post infection.

**Figure 5:**
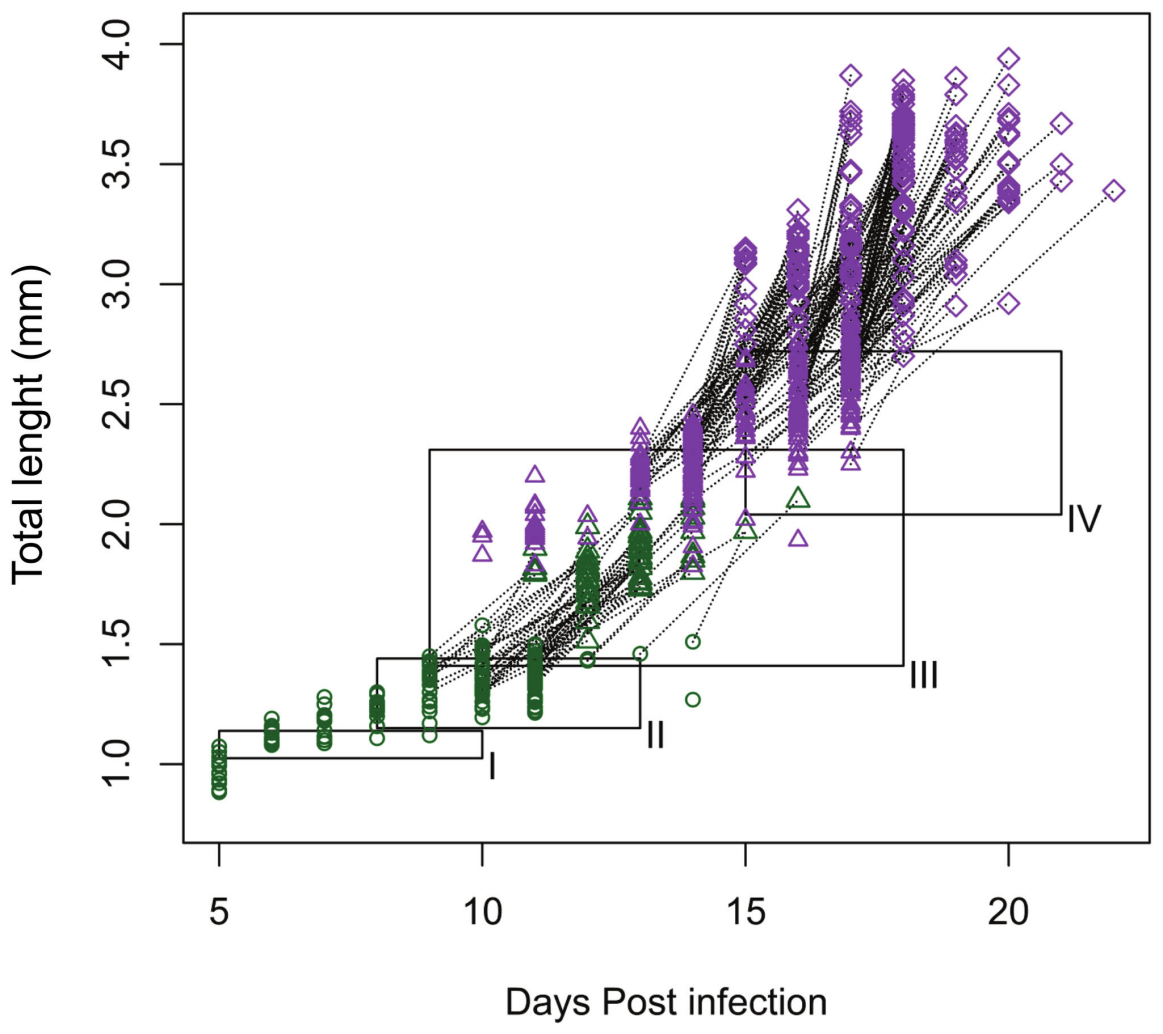
Size measurements for *L* *. salmonis* larvae incubated in hatching wells. Pre-incubation measurements for incubated samples are shown as blue circles (chalimus I/II category) and red triangles (chalimus III/IV category). The post-incubation measurements are shown as blue triangles (chalimus III-IV category) and red diamonds (preadults). For molted individuals the pre- and post-incubation measurements are connected with a dotted line. Frames I-IV represent the sizes for the chalimus I-IV stages as reported by Schram [19] and durations as reported by Johnson and Albright [28].

In experiment 1 a total of 198 *L* *. salmonis* , sampled daily from fish in the period from 6-14 DPI, were assigned to the chalimus I/II category and incubated. Among these 48 molts were observed (exuvia present, example shown in Figure 1), all of which molted to the chalimus III/IV category in the incubators between 10-16 DPI (Figures 4 and 5). Among the 427 larvae sampled from 10 to 17 DPI assigned to the chalimus III/IV category a total of 154 molts were observed, all of which molted to the preadult 1 stage between 14-22 DPI. Of the 255 chalimus larvae incubated in experiment 2, a total of 71 lice were defined as having molted (TL>15% during incubation). In experiment 2, as in experiment1, all larvae in the chalimus I/II category that molted were post molt identified as belonging to the chalimus III/IV category (n=30) and all the larvae in the chalimus III/IV category that molted were post molt identified as preadult 1 larvae (n=41). None of the chalimi under observation in incubators in either experiment molted into the morphological category/cluster they were assigned to before incubation.

### Development rate

A total of 154 (Experiment 1) and 41 (Experiment 2) chalimi that molted in hatching wells had their sex attributed according to the sex of corresponding post-molt preadults. The preincubation samples show that females of the last chalimus stage generally were larger and molted later than their male counterparts (Figure 2A-C). This observation is consistent with the dominance of males (98%) among the preadults sampled on days 14-16 in experiment 2 (Figure 2A-C). The earliest chalimi were sampled at 5 DPI from a population of 40% copepodids and 60% chalimi. At 13 DPI 50% of the molts from the chalimus I/II category had taken place (Figures 4 and 5). At 17 and 19 DPI 50% of the molts from the chalimus III/IV category males and females respectively had been observed. Under the questionable assumption that males and females enter the chalimus I/II stage simultaneously the average chalimus life span was 12 days for males and 14 days for females.

## Discussion

The ecological and economic importance of the salmon louse has stimulated in a large body of research on this species [21,22,23,24,25,26,27] and it might have been expected that the correct life cycle had been well established. However, the validity of the salmon louse life cycle has been questioned [2]. To bring clarity to this subject we therefore evaluated the commonly accepted life cycle model comprising ten stages including four chalimus stages [8,19,28] and an alternative life cycle model comprising eight stages including two chalimus stages as proposed by Ohtsuka et al. [2].

Morphological clustering shows that assigning the chalimi to two chalimus clusters explains the more than 87% of variability in the data (TL,CL and CW), whereas addition of further stages does not appreciably increase the amount of variation explained. This suggests that chalimi should be divided into two groups based on morphology and that *L* *. salmonis* has only two chalimus stages, particularly when considering the fact that development in size (TL, CL and CW) within chalimus clusters 1 and 2 shows no sign of sudden increase as would be expected if within-cluster size variability were a result of molting. Obtaining a definitive answer on the number of chalimus stages, however, relies on observation of molting events. A total of 272 molting events observed in incubators unambiguously showed that larvae assigned to the chalimus I/II category molted exclusively to chalimus III/IV category, and that all larvae assigned to the chalimus III/IV category molted to preadults only. There were no records of molts within the chalimus I/II category, as would be expected if chalimus I and II were separate stages as defined by Johnson and Albright [8] and Schram [19]. Also, there were no records of molts within the chalimus III/IV category as would be expected if chalimus III and chalimus IV were separate stages. Robust daily samples and incubation of chalimi exclude the possibility that molts were missed. The present data are incompatible with a salmon louse life cycle model comprising four chalimus stages separated by molts as suggested by Johnson and Albright [8]. The results are in accordance with a life cycle comprising 2 chalimus stages as hypothesized by Ohtsuka et al. [2]. Using the same kind of segmental setation pattern observations and reasoning applied by Ohtsuka et al. [2], this hypothesis has been further strengthened in a study of *L* *. elegans* [16]. However, Venmathi Maran et al. [16] did not present any new evidence for the hypothesis and eliminated two chalimi stages based only on antennule setal counts, without observing the molting process. We supply the necessary experimental evidence and conclude that the life cycle of *Lepeophtheirus* *salmonis* has 2 chalimus stages and consequently only 6 copepodid stages as is the case of all other members of the subclass Copepoda [2] for which the life cycle is known. The life cycle thus comprises 8 developmental stages; nauplius 1 and 2, copepodid, chalimus 1 and 2, preadult 1 and 2 and the adult stage, and this terminology will be used from now on. In the light of arguments presented by Ohtsuka et al. [2] and the present results, it is also likely that *L* *. pectoralis* [9], *L* *. dissimulatus* [7], *L* *. hospitalis* [10] and other *Lepeophtheirus* spp. have only two chalimus stages in their life cycle, as has also been suggested for *L* *. elegans* [16].

The temporal increase in TL appeared to be continuous save where interrupted by two sudden increments, explained by molting events, around 11 and 16 DPI suggesting a significant intramolt growth in length. In contrast CL and CW appear more stable between the sudden size increments around 11 and 16 DPI. The continuous increase in TL and the more stable CL and CW between the molting events around 11 and 16 DPI are not unexpected as intra-molt growth in *L* *. salmonis* adults has previously been reported to result from abdominal growth whereas cephalothorax lengths and widths were stable [29]. The results indicate that female chalimus 2 are larger and older than their male counterparts when they molt into preadults. This would imply that males are smaller and develop faster than females, which is in accordance with our observations and previous reports [8,19,30]. The slight temporal increase in CL and CW evident between the discrete increments could be caused by intramolt growth, an increasing recruitment of larger, possibly predominantly female, individuals (molting from the preceding stage) and a continuous loss of the smaller, possibly mostly male, individuals (molting into the next stage), or a combination of these three factors. Hence the previously identified chalimus I and II stages (Johnson and Albright, 1991b) are likely to represent young and old chalimus 1 whereas the previously identified chalimus III and IV stages represent young and old chalimus 2. Regardless of the driving force behind the gradual intramolt increase in TL, CL and CW, the results show that the reported size differences between the former chalimus I and II on one hand, and the former chalimus III and IV on the other, is convincingly explained by intramolt size increase as suggested by Ohtsuka et al. [2] and sexual size dimorphism [16]. Details of chalimus growth and sexual size dimorphism in *L* *. salmonis* will be addressed thoroughly in an ongoing study at SLRC.

The salmon louse is a major pest in salmonid aquaculture in the Northern Hemisphere [1] and there are also concerns over the transfer of lice between farmed and wild salmon [22]. The large economical and environmental effects have resulted in considerable efforts to control *L* *. salmonis* , mainly using chemotherapeutants with the unfortunate side effect of resistance development against all available drugs except molt inhibitors such as diflubenzuron and teflubenzuron. Although molt inhibitors have not been widely used it is clearly important to know the correct number of molts in the life cycle in order to be able to utilize such pesticides effectively. The corrected lifecycle represent important knowledge for future research and salmon louse pest management.
